# IMPROVING COMFORT LEVELS IN PERFORMING AWAKE FIBEROPTIC INTUBATION: A COLLABORATIVE EFFORT BETWEEN OTOLARYNGOLOGY AND ANESTHESIA

**DOI:** 10.51894/001c.122884

**Published:** 2024-08-30

**Authors:** Benjamin Gillette, Michael Toscano, Michael Owens, Gary Kwartowitz

**Affiliations:** 1 Ear, Nose, and Throat (ENT) McLaren Oakland

## 31

### INTRODUCTION

Awake fiberoptic intubation (AFI) is considered the “gold standard” for a difficult airway where relaxation may lead to a “cannot ventilate, cannot intubate” situation. Otolaryngology/Anesthesia departments often work in conjunction when performing AFI. Many institutions have slight variations in protocols, which can create confusion when performing AFI. Having a standardized protocol has the potential to reduce errors.

### AIMS/OBJECTIVES

The aim of this study was to implement a standardized AFI protocol, and to measure Resident/attending comfort level in performing AFI pre and post implementation of the protocol.

### METHODS

This study was conducted from September 2022 to September 2024. An initial Zoom presentation was conducted amongst Otolaryngology/Anesthesia departments at a single institution, which involved creating a collaborative standardized protocol combining procedural from both departments. A survey inquiring about comfort levels in performing AFI amongst Otolaryngology/Anesthesia Residents/attendings was distributed pre implementation of the protocol, 6 months post implementation and 10 months post implementation. Outcome measures included demographic data, comfort level in performing AFI and a comments section for why the protocol is or is not helpful.

### RESULTS

There was a total of 54 respondents amongst Otolaryngology/Anesthesia Residents/attendings. Respondents were asked if they felt less, equally, or more comfortable 6 months after implementation of the protocol and results were 6%, 59% and 35%; respectively. Respondents were then asked this again 10 months post implementation and results were 0%, 53% and 47%; respectively. A comments section was also included in the survey and notable comments included: “would appreciate a hands-on learning session,” “having a protocol reduces errors,” and “the protocol adds consistency amongst providers.”

### CONCLUSIONS

Creating a standardized AFI protocol increased overall comfort levels in performing AFI amongst Otolaryngology/Anesthesia Residents/attendings at this single institution. A standardized protocol is simple to implement and has the potential to increase comfort levels and reduce errors when performing AFI

